# Functional Consequences of the Macrophage Stimulating Protein 689C Inflammatory Bowel Disease Risk Allele

**DOI:** 10.1371/journal.pone.0083958

**Published:** 2013-12-23

**Authors:** Steven E. Kauder, Lydia Santell, Elaine Mai, Lilyan Y. Wright, Elizabeth Luis, Elsa N. N'Diaye, Jeff Lutman, Navneet Ratti, Susan M. Sa, Henry R. Maun, Eric Stefanich, Lino C. Gonzalez, Robert R. Graham, Lauri Diehl, William A. Faubion, Mary E. Keir, Judy Young, Amitabha Chaudhuri, Robert A. Lazarus, Jackson G. Egen

**Affiliations:** 1 Discovery Immunology, Genentech Inc., South San Francisco, California, United States of America; 2 Early Discovery Biochemistry, Genentech Inc., South San Francisco, California, United States of America; 3 Biochemical and Cellular Pharmacology, Genentech Inc., South San Francisco, California, United States of America; 4 Immunology, Tissue Growth and Repair- Diagnostics Discovery, Genentech Inc., South San Francisco, California, United States of America; 5 Protein Chemistry, Genentech Inc., South San Francisco, California, United States of America; 6 Pharmacokinetics and Pharmacodynamics, Genentech Inc., South San Francisco, California, United States of America; 7 Pathology, Genentech Inc., South San Francisco, California, United States of America; 8 Immunology, Tissue Growth and Repair -Human Genetics, Genentech Inc., South San Francisco, California, United States of America; 9 Molecular Oncology, Genentech Inc., South San Francisco, California, United States of America; 10 Division of Gastroenterology and Hepatology, Mayo Clinic, Rochester, Minnesota, United States of America; INSERM, France

## Abstract

**Background:**

Macrophage stimulating protein (MSP) is a serum growth factor that binds to and activates the receptor tyrosine kinase, Recepteur d'Origine Nantais (RON). A non-synonymous coding variant in MSP (689C) has been associated with genetic susceptibility to both Crohn's disease and ulcerative colitis, two major types of inflammatory bowel disease (IBD) characterized by chronic inflammation of the digestive tract. We investigated the consequences of this polymorphism for MSP-RON pathway activity and IBD pathogenesis.

**Methods:**

RON expression patterns were examined on mouse and human cells and tissues under normal and disease conditions to identify cell types regulated by MSP-RON. Recombinant MSP variants were tested for their ability to bind and stimulate RON and undergo proteolytic activation. MSP concentrations were quantified in the serum of individuals carrying the MSP 689R and 689C alleles.

**Results:**

In intestinal tissue, RON was primarily expressed by epithelial cells under normal and disease conditions. The 689C polymorphism had no impact on the ability of MSP to bind to or signal through RON. In a cohort of normal individuals and IBD patients, carriers of the 689C polymorphism had lower concentrations of MSP in their serum.

**Conclusions:**

By reducing the quantities of circulating MSP, the 689C polymorphism, or a variant in linkage disequilibrium with this polymorphism, may impact RON ligand availability and thus receptor activity. Given the known functions of RON in regulating wound healing and our analysis of RON expression patterns in human intestinal tissue, these data suggest that decreased RON activity may impact the efficiency of epithelial repair and thus underlie the increased IBD susceptibility associated with the MSP 689C allele.

## Introduction

Crohn's disease (CD) and ulcerative colitis (UC) are two clinically distinct forms of inflammatory bowel disease (IBD) characterized by chronic inflammatory responses within the intestinal tract. Genome-wide association studies (GWAS) examining the frequencies of single nucleotide polymorphisms (SNPs) in CD and UC patients have identified a large number of shared and unique susceptibility alleles. While individually, the majority of these loci contribute only modestly to the overall genetic risk in IBD, they converge on several common biological pathways, such as autophagy, T helper 17 differentiation/activation, innate immune cell activation, and epithelial wound repair, revealing a complex picture of the cellular and molecular processes involved in the pathogenesis of these diseases [1]. A major difficulty in using GWAS data to infer disease-relevant biology and/or identify therapeutic targets is the lack of information on the identity of causative alleles associated with susceptibility loci and the effect that these genetic variations have on specific biological processes.

Using both GWAS and candidate gene approaches, several groups have identified polymorphisms in macrophage stimulating protein (MSP, also known as hepatocyte growth factor-like (HGFL) and MST1) that are associated with susceptibility to both CD and UC [2]–[8]. The allele thought to be responsible for this increased genetic risk is a non-synonymous coding SNP in MSP (rs3197999) that leads to an arginine to cysteine change at amino acid 689 (689R to 689C) [7]. MSP is a plasminogen-related soluble serum growth factor expressed by the liver and secreted into the blood as an inactive single-chain protein (pro-MSP) incapable of binding its receptor, the Met-family receptor tyrosine kinase, Recepteur d'Origine Nantais (RON, also known as MST1R). Proteolytic cleavage of pro-MSP between R483 and V484 converts it into an active, two-chain disulfide-linked α/β heterodimer able to bind RON with high affinity and induce receptor signaling [9]–[11]. pro-MSP can be proteolytically activated by a number of different serine proteases, including those involved in the coagulation cascade and induced during inflammatory responses [12]–[15]. Thus, cleavage of pro-MSP at sites of tissue damage leads to local activation of RON.

RON expression has been reported in epithelial cells, subsets of macrophages, neuroendocrine tissues, and developing bone [9], [16]–[18]. In the context of epithelial cell biology, MSP signaling through RON leads to induction of proliferation, survival, migration, and extracellular matrix adherence [9], [10], [19]–[21]. As such, RON signaling has been suggested to drive both epithelial wound healing and oncogenesis of epithelial-derived tumors [14], [22], [23]. More recently, the MSP-RON pathway has been proposed as a key negative regulator of inflammatory responses by suppressing production of pro-inflammatory factors from macrophages [24]–[32]. These studies have lead to speculation that the increased genetic risk for IBD associated with the MSP 689C polymorphism is a direct consequence of defects in RON-mediated inhibition of macrophage activation [7], [33]. However, the expression of RON on human macrophage subsets and its role in regulating their activity has not been thoroughly examined.

The functional consequences of the 689C polymorphism for MSP-RON pathway activity have only recently begun to be examined and remain unclear. Based on its suspected proximity to the putative RON binding site, the polymorphism was originally hypothesized to alter binding between MSP and RON [7]. Consistent with this hypothesis, a recent study has suggested that the 689C polymorphism reduces MSP binding affinity to RON. While the impact of this affinity difference on the *in vitro* or i*n vivo* cellular activities of MSP was not assessed, the authors speculated that impairment of MSP-mediated suppression of macrophage activation would lead to exacerbated inflammatory responses within carriers of the polymorphism [34]. Paradoxically, a more recent study found that the 689C polymorphism increases MSP stimulatory activity in a human monocytic cell line, inducing greater *in vitro* migration and proliferation. The underlying biochemical or structural mechanisms responsible for this increased activity were not assessed [35].

Here, we investigate the biochemical and functional consequences of the 689C polymorphism in the context of RON signaling and IBD pathogenesis. We demonstrate that in humans, RON is minimally expressed by intestinal macrophage populations and is instead restricted to epithelial compartments, both under normal conditions and within the context of IBD. Furthermore, we find no effect of the 689C polymorphism on MSP-RON binding affinity or kinetics, MSP-dependent RON signaling, or pro-MSP proteolytic activation. Rather, in a large collection of matched serum and DNA samples from normal individuals and IBD patients, we demonstrate that the 689C polymorphism is associated with decreased MSP serum concentrations. These data expand our understanding of the mechanism by which the 689C polymorphism regulates MSP function, implicate RON expression on epithelial cells as being critical for maintenance of intestinal homeostasis, and provide further evidence that alterations in specific tissue repair pathways can mediate genetic susceptibility to IBD.

## Materials and Methods

### Ethics statement and Human samples

Human intestinal resection samples (Table S1) were obtained through a collaboration with the Mayo Clinic (Rochester, Minnesota) that was reviewed and approved by the Mayo Clinic Institutional Review Board. Matched serum, DNA, and intestinal biopsy RNA samples were collected as part of the multi-center EMBARK observational clinical trial in IBD sponsored by Genentech [36]. Written informed consent was obtained from all patients.

### Animal studies

C57Bl/6 animals from Jackson Laboratories were used for all animal studies. All animal experiments were approved by the Genentech Institutional Animal Care and Use Committee.

### Cloning, expression, and purification of recombinant proteins

Recombinant human pro-MSP 689R or 689C (Q19-G711; C672A), MSPβ 689R or 689C (V484-G711; C588S, with and without C672A) were cloned into pRK expression vectors with C-terminal 6-His tags. An R483E mutation was introduced into the P1 position of the pro-MSP cleavage site to make scMSP 689R or 689C (Q19-G711; R483E, C672A) as noncleavable versions of single-chain pro-MSP (scMSP). All mutations were made with the *QuikChange II XL* site-directed mutagenesis kit (Agilent Technologies). Chinese hamster ovary (CHO) cells were transiently transfected with expression vectors. Secreted proteins were purified by Ni-NTA affinity chromatography followed by size exclusion chromatography on Superdex 200 10/300 GL or Superdex 75 10/300 GL to isolate monomeric proteins based on their elution profile (GE Healthcare).

Recombinant human RON comprising the Sema, PSI and IPT1 domains (E25-M682) was made as an Fc fusion with human IgG1 (RON-Fc) by expressing in CHO cells. RON-Fc was purified by affinity chromatography on a MabSelect Sure column (GE Healthcare) followed by size exclusion chromatography (Superdex 200 10/300 GL). Protein was purified in HEPES Buffered Saline (HBS) and stored at −20°C. Recombinant human RON Sema/PSI (residues E25-P568) an N-terminal gp67 secretion signal and containing a C-terminal His_6_-tag was cloned into the Gateway vector pENTR/D-TOPO (Life Technologies). Subcloning into the pDEST-8 vector in combination with the Bac-to-Bac system (Life Technologies) was used to generate recombinant baculovirus according to manufacturers protocol. *Trichoplusia ni* insect cells (1×10^6^ cells per ml) were infected with recombinant baculovirus with a multiplicity of infection of three in ESF921 medium (Expression Systems). After a 72 h incubation, RON Sema/PSI was purified from the media by Ni-NTA affinity chromatography followed by size exclusion chromatography on Superdex 200 10/300 GL (GE Healthcare) in 20 mM HEPES pH 7.2 and 150 mM NaCl (HBS) and stored at −20°C.

The extracellular domain of the human recombinant hepsin harboring a C-terminal His_8_-tag (sHepsin) was expressed and purified as described [15]. Antibody25 (Fab25) inhibits hepsin enzymatic activity was generated by using antibody phage display and subsequently expressed in *E. coli* and purified as described previously [37].

### Activation of pro-MSP by sHepsin

To produce active pure two-chain MSP for studies herein, pro-MSP was incubated with sHepsin at a 1∶100 ratio in activation buffer (50 mM Tris-HCl, pH 8.0, 150 mM NaCl, 0.05% CHAPS) overnight at room temperature. sHepsin was removed by adding a 10-fold molar excess of anti-hepsin (Fab25) followed by Protein A-Sepharose Chromatography (GE Healthcare). This resulted in complete conversion of pro-MSP to the active two-chain form. To determine activation kinetics, pro-MSP (100 µg/ml, 1.25 µM) was incubated 1 h at 37°C with various concentrations (100 nM–97 pM, 2-fold dilution series) of sHepsin. The reaction was stopped by adding SDS-PAGE sample buffer followed by electrophoretic analysis on 4%–20% Tris-glycine gels (Life Technologies) under reducing conditions.

### Cell isolation, differentiation, and culture

Cell lines were cultured according to standard protocols. Parental 3T3 and A2780 cells were stably transfected with human RON to generate 3T3-hRON and A2780-hRON cells respectively [38], for which medium was supplemented with 400 µg/ml G418. HPC5 human primary colon cells (Celprogen) were maintained in colon cell culture medium (Celprogen).

Human peripheral blood mononuclear cells (PBMCs) were isolated from blood by density gradient centrifugation with Ficoll-Paque PLUS (GE Healthcare). Monocytes were purified from PBMCs with the Monocyte Isolation Kit II (Miltenyi Biotec). Human monocyte-derived macrophages were obtained by incubation of CD14^+^ monocytes for one week in RPMI supplemented with 20% fetal bovine serum (FBS), 100 U/ml penicillin, 100 µg/ml streptomycin, 2 mM L-glutamine, and 100 ng/ml M-CSF (R&D Systems). Macrophages were treated with 100 ng/ml LPS and 20 ng/ml IFN-γ or 20 ng/ml IL-4 for 18 h in RPMI plus 5% FBS, or with 100 ng/ml LPS on IgG-coated plates for 24 h in RPMI plus 20% FBS.

Single cell suspensions of resected intestinal tissues were prepared by dissecting out serosa, lamina muscularis, and submucosa, leaving approximately 3 g of tissue including lamina propria and epithelium. This was incubated for 15 min at 30°C on an orbital shaker in 50 ml HBSS with 5 mM DTT, then in 50 ml HBSS with 1 mM EDTA. Tissue was washed twice in 50 ml RPMI plus 10% FBS, minced into 0.5 cm pieces, and digested 20 min in 50 ml RPMI, 10% FBS, 1.5 mg/ml collagenase VIII (Sigma-Aldrich), 0.1 mg/ml DNase I at 37°C on an orbital shaker. Digested tissue was filtered through a 70 µm pore filter, washed, and resuspended in FACS buffer (PBS plus 2% FBS). For leukocyte purification, cells were pelleted, resuspended in 7 ml isotonic 35% Percoll (GE Healthcare), underlayed with 6 ml isotonic 60% Percoll, and centrifuged 20 min at 2000 RPM at 4°C. Cells at the Percoll interface were collected, washed, and resuspended in FACS buffer.

Murine bone marrow derived macrophages were obtained by incubating unfractionated bone marrow cells in DMEM plus 10% FBS, 100 U/ml penicillin, 100 µg/ml streptomycin, 2 mM L-glutamine, and 30% conditioned media from L929 cells. Fresh growth media was added on day 3 of the culture and adherent macrophages were harvested on day 7. Cells were uniformly F4/80 and CD11b positive at this time point.

Peritoneal exudate cells were harvested from mice under steady-state conditions or 4 days after intraperitonal injection with 1 ml of 3% thioglycollate by peritoneal lavage with 9 ml of RPMI. Cells were washed once with RPMI and resuspended in FACS buffer. Liver mononuclear cells were obtained as described [39], with some modification. Briefly, livers were perfused through the portal vein with 3 ml of digest buffer consisting of RPMI plus 0.2 mg/ml of Liberase TL and 0.1 mg/ml DNase I (Roche Applied Science). Livers were excised and further incubated in digest buffer for 40 min at 37°C. After manual disruption by repeated pipetting, liver cell suspensions were washed in Hanks' balanced salt solution (HBSS) (Life Technologies), resuspended in 35% Percoll, and centrifuged at 800×g for 20 min, collecting the cell pellet. Red blood cells were lysed with Ack lysis buffer (Lonza) and cells were resuspended in FACS buffer. For single cell suspensions of murine colon, colons were removed from the animals, flushed with HBSS, cut into 2 cm pieces, and incubated in HBSS containing EDTA and DTT for 15 min at 37°C with constant shaking. This incubation period was kept short to avoid loss of epithelial cells. The tissue was washed 2 times in RPMI, minced, and incubated in RPMI plus 0.2 mg/ml of Liberase TL and 0.1 mg/ml DNase I for 20 min at 37°C with constant shaking. The suspension, containing both mononuclear cells and epithelial cells, was filtered through 100 µm and 70 µm filters, washed, and resuspended in FACS buffer.

### Flow cytometry

Murine cells were stained with fluorochrome-labeled antibodies against F4/80 (clone BM8), CD11b (clone M1/70), MHC class II (clone M5/114.15.2), and either mouse RON (clone PH4, Genentech, Inc.) or a murine IgG2a isotype control antibody (Genentech, Inc). Human cells were stained with fluorochrome-labelled antibodies against CD14 (clone 61D3), CD16 (clone 3G8), CD45 (clone HI30) EpCAM (clone VU1D9), MHC class II (LN3), and either human RON (Clone 1A2.2, Genentech, Inc.) or a murine IgG2a isotype control antibody (Genentech, Inc). Unless otherwise noted, antibodies were purchased from Abcam, BD Biosciences, Biolegend, or eBioscience. *Ex vivo* analyzed murine and human cells were stained with a LIVE/DEAD Aqua or Violet viability stain (Life Technologies). Cells were analyzed on a LSRII flow cytometer (BD Biosciences). Live cells were identified based on size and the viability stain and RON expression was determined by gating on the indicated population of cells using Flowjo software (Treestar).

### MSP binding to plate immobilized RON

MaxiSorp plates (Nalge Nunc International) were coated overnight at 4°C with 2 µg/ml of rabbit anti-human IgG Fc specific antibody (Jackson ImmunoResearch Laboratories) in 50 mM sodium carbonate buffer, pH 9.6. After blocking with assay buffer (50 mM HEPES pH 7.2, 150 mM NaCl, 5 mM CaCl_2_, 1% BSA and 0.1% Tween-20), 1 µg/ml RON-Fc fusion protein in assay buffer was added and plates were incubated for 1 h with gentle shaking at room temperature. After washing with PBS + 0.05% Tween-20, MSP (1000 nM-0.2 pM, 2-fold dilution series) was added for 1 h. Bound MSP was detected using anti-His-HRP (Qiagen Inc.) followed by addition of TMB/H_2_O_2_ substrate (Thermo Scientific). The reaction was stopped with 1M H_3_PO_4_ and the absorbance at 450 nm (OD_450 nm_) was measured on a SpectraMax Plus^384^ plate reader (Molecular Devices, LLC). The half maximal effective concentration of MSP (EC_50_) was determined by a 4-parameter fit using Kaleidagraph (Synergy Software).

### Kinetic measurement of MSP binding to RON by surface plasmon resonance (SPR)

Binding affinities of MSP, MSP β or scMSP to RON were determined using a Biacore 3000 optical biosensor equipped with a research-grade CM5 sensor chip (GE Healthcare). Amine coupling reagents, N-ethyl-N′-dimethylamino-propylcarbodiimide (EDC), N-hydroxy-succinimide (NHS) and sodium ethanolamine-HCl, pH 8.5, were obtained from GE Healthcare. Standard coupling protocols were used to immobilize RON Sema/PSI onto the biosensor surface. A biosensor chip that was subjected to the amine coupling procedure with no coupled protein was used to correct for non-specific MSP binding. To determine MSP binding kinetics, between 50 and 100 response units (RU) were captured on each of three immobilized anti-human Fc surfaces. MSP, MSP β or scMSP (500–3.9 nM, 2 fold dilution series) were injected in HBS-P buffer (10 mM HEPES, pH 7.5, 150 mM NaCl, 0.005% P20) at 25°C with a flow rate of 30 µl/min and dissociation monitored for 4 min. Between measurements, the biosensor surfaces were regenerated with a 120 s pulse of 10 mM glycine-HCl pH 1.5 followed by a 2 min wash with running buffer. Each data set was fit globally to a simple one-to-one Langmuir binding model (BIA evaluation 4.1, GE Healthcare) to determine the kinetic parameters k_on_ and k_off_. The equilibrium dissociation constants (K_D_) were then calculated as a ratio (k_off_/k_on_) of these rate constants.

### Kinetic measurements of MSP binding to RON by biolayer interferometry (BLI)

Real-time kinetic measurements of MSP binding to RON were conducted using an Octet RED384 (ForteBio, Inc). Samples or buffer were dispensed into 96-well microtiter plates (Greiner Bio-One North America, Inc.) at a volume of 200 µl per well. Anti-human IgG Fc Biosensors were used for the experiments. Each experiment consisted of three steps: incubation with ForteBio Kinetic buffer diluted 1∶10 in PBS assay buffer to establish an equilibrium for 120 s (baseline), incubation with 10 µg/ml RON-Fc in assay buffer to coat the biosensor with binding target for 900 s (load), incubation with MSP, MSP β or scMSP in assay buffer containing various concentrations (75–4.7 nM, 2-fold dilution series) for 400 s (association), and incubation with assay buffer to measure the off-rate of MSP for 400 s (dissociation). Operating temperature was maintained at 30°C. Data were generated automatically by the Octet Data Acquisition 7.0 software. Each data set was fitted globally to a simple one-to-one binding model using ForteBio Analysis Software 7.0 to determine the kinetic parameters k_on_ and k_off_. The equilibrium dissociation constants (**K**_D_) were then calculated as a ratio (k_off_/k_on_) of these rate constants.

### Equilibrium measurement of MSP binding to RON by radioligand binding assay

MSP proteins were iodinated using the Iodogen method (Thermo Scientific) and purified from free Na^125^I by gel filtration using a NAP-5 column. Specific activities ranged from 12.26 to 18.65 µCi/μg. Competition reaction mixtures were made of a fixed concentration of iodinated MSP and unlabeled MSP serially diluted 1- to 2-fold ten times starting at 5 µM in a volume of 50 µl. 3T3-hRon cells were washed with binding buffer, consisting of DMEM with 1% bovine serum albumin, 325 nM human IgG, 50 mM HEPES (pH 7.2) and 0.1% sodium azide. 150,000 cells in 0.2 ml binding buffer were added to competition reaction mixtures. Competition reactions with cells were incubated for 2 h at room temperature and transferred to a Multiscreen filter plate (MIllipore) and washed 4 times with binding buffer. Filters were counted on a Wallac Wizard 1470 gamma counter (PerkinElmer Life and Analytical Sciences). Binding affinities were determined using NewLigand software (Genentech, Inc) [40].

### Western blotting

A2780-hRON and BxPC3 cells were grown overnight in media containing 0.5% BSA. Cells were treated with 100 ng/ml MSP 689R, MSP 689C or nothing for 20 min at 37°C. Samples were prepared by washing the cell monolayer twice with ice cold PBS followed by the addition of SDS-PAGE sample buffer and quantified by BCA Protein Assay (Thermo Scientific, Rockford, IL). Proteins (20 µg) were electrophoresed on 4%–20% Tris-glycine gels (Life Technologies), transferred to nitrocellulose membranes and blocked with Odyssey blocking buffer (Li-Cor Biosciences) for 1 h at room temperature. Membrane was probed with Akt Polyclonal Antibody (Cell Signaling Technology), and Phospho-Akt Monoclonal Antibody, Ser473 (Cell Signaling Technology) overnight at 4°C. After washing, the membrane was incubated with IRDye™800 conjugated goat anti-mouse IgG (Rockland Immunochemicals) and AlexaFluor 680 goat anti-rabbit IgG (Life Technologies) for 1 h. The amount of phosphorylated and total kinase expression was detected using the Odyssey Infrared Imaging System (Li-Cor Biosciences).

### MSD analysis of RON signaling

3T3-hRON cells were seeded in DMEM with 0.5% bovine calf serum (BCS). HCT15 and HPC5 cells were seeded in RPMI with 0.5% FBS. The next day, 3T3-hRON cells were treated with titrations of MSP 689R, MSP 689C, scMSP (250 nM to 4 pM, 1.5-fold dilution series in medium) or medium alone and HCT15 and HPC5 cells were treated with titrations of MSP 689R, MSP 689C (250 nM to 3.2 pM, 5-fold dilution series in medium) or medium alone. After 30 min cells were lysed in MSD lysis buffer (Meso Scale Discovery). For detection of Akt phosphorylation, lysates were added to MULTI-SPOT 96-Well 4-Spot Phospho(Ser473)/Total Akt plates (Meso Scale Discovery) which were incubated according to manufacturers instructions and read in a SECTOR Imager 6000 (Meso Scale Discovery).

### Immunohistochemistry and in situ hybridization on fixed tissue sections

For IHC of human tissues, 4 µm sections were cut from formalin fixed, paraffin-embedded intestinal tissue. Staining was performed on the Ventana Discovery XT Autostainer platform (Ventana Medical Systems). Deparaffinization, endogenous peroxidase blocking as well as pretreatment using CC1 standard antigen retrieval was performed using Ventana ready to use reagents. Goat polyclonal anti-human RON antibody (R&D Systems) was then diluted to 0.5 µg/ml in 3% BSA/PBS and sections were incubated for 32 min at 37°C. Sections were subsequently incubated with an unconjugated rabbit-anti-goat secondary linker antibody (Vector Labs) followed by an anti-rabbit-OMNIMAP-HRP kit (Ventana Medical Systems) and ChromoMap DAB colorimetric reagents (Ventana Medical Systems). Slides were counterstained with hematoxylin (Ventana Medical Systems) and dehydrated, cleared and mounted for viewing.

For IHC on murine tissues, 4 µm sections were cut from formalin fixed, paraffin-embedded intestinal tissues. Sections were deparaffinized in xylenes and rehydrated through a graded series of alcohols. Sections were then pre-treated for antigen retrieval using Target Retrieval Solution (DAKO). Sections were blocked for endogenous peroxidase activity using KPL blocking solution (KPL, Inc.), for avidin/biotin using an avidin/biotin blocking kit (Vector Labs), and for IgG binding with TNB Blocking buffer (Perkin Elmer). Sections were incubated overnight at 4°C with anti-murine RON goat polyclonal antibody (R&D Systems) at 2.5 µg/ml. Sections were then incubated with a biotinylated donkey-anti-goat secondary antibody (Jackson ImmunoResearch Laboratories) followed by ABC-HRP Elite reagents (Vector Labs). Chromogenic development was accomplished using a metal enhanced DAB colorimetric peroxidase substrate (Thermo Scientific). Sections were then counterstained with Myer's Hematoxylin (Rowley Biochemical Institute), dehydrated, cleared with xylenes and mounted for viewing.

Non-isotopic ISH was performed on 4 µm FFPE sections using QuantiGene® ViewRNA ISH Tissue Assay (Affymetrix/Panomics) following the manufacturer's protocol, using chromogenic detection with Fast Red substrate. Gene-specific probe set for human RON (VA1-13401), target region 1541–2724 in Genbank accession NM_002447, was used to detect expression of RON in colon samples, with a probe set to *Bacillus subtilis* dihydropicolinate reductase (dapB) (VF1-11712) used as a negative control. Prior to testing for RON message, mRNA presence in colon samples was confirmed by isotopic ISH using ^33^P-UTP labeled anti-sense riboprobes specific for human β-actin [41].

### *In vitro* scratch wound assay

Parental 3T3 or 3T3-hRON cells were suspended in DMEM with 0.5% BCS and seeded in collagen-coated 96 well plates. The next day, scratch wounds were made in each well using the WoundMaker 96 (Essen Bioscience). After two washes in PBS, medium alone or medium containing 15 nM MSP 689R, MSP 689C, or scMSP was added to the wells. Cells were incubated 39 h in the IncuCyte Imaging System (Essen Bioscience) with imaging every 3 h. Using the IncuCyte software package (Essen Bioscience), cell density within the wound was calculated relative to density outside the wound. Images were uniformly processed post-analysis with a Gaussian filter, contrast enhancement, and shadowing using ImageJ [42] (National Institutes of Health) to visualize cell borders.

### Quantitative PCR and RT-PCR

To determine the rs3197999 genotype of EMBARK cohort members, 10 ng of genomic DNA was used in the Taqman SNP Genotyping Assay (Applied Biosystems). For analysis of RON mRNA expression in epithelial cell lines and myeloid cells, total RNA was isolated with the RNeasy kit with on-column DNase digestion (Qiagen). 80 ng RNA was assayed with the Quantitect Probe RT-PCR kit (Qiagen), and RON expression was normalized to RPL19 expression using the ΔC_T_ method. For RON amplification, primers 5′-AGGGCAGTCCTGCAACAT-3′, 5′-GAGTCCACTGTGCCCAGAA-3′, and taqman probe 5′- ACAGGGTCCACAGCAGGCACTC-3′ were used. For RPL19 amplification, primers 5′-CAATGCCAACTCCCGTCAG-3′, 5′-GTCACAGGCTTGCGGATGA-3′, and taqman probe 5′-AGATCCGGAAGCTCATCAAAGATGGGCT-3′ were used. Reactions were run on the ABI 7500 Real Time PCR system and analyzed with the 7500 Software (Applied Biosystems). For analysis of mRNA expression in intestinal biopsies, tissue samples were homogenized with 3 mm using a TissueLyzer (Qiagen) and RNA isolated using the RNeasy kit (Qiagen). RNA integrity was assessed with the Agilent 2100 Bioanalyzer using the Agilent RNA 6000 Pico Kit (Agilent Technologies). Reactions were run on the BioMark HD System (Fluidigm) using human RON primer set Hs00899925_m1 (Applied Biosystems) and GAPDH primer set Hs99999905_m1 (Applied Biosystems). RON expression was normalized to GAPDH and a reference human RNA sample using the ΔΔC_T_ method.

### ELISA for MSP levels in human serum

For assays of human serum, MaxiSorp plates were coated overnight at 4°C with 1 µg/ml of anti-hMSP α antibody (R&D Systems) in 0.05 M carbonate/bicarbonate buffer, pH 9.6, then incubated 1 h at room temperature in blocking buffer (PBS, 0.5% BSA, 15 ppm Proclin pH 7.4), and washed with 100 µl washing buffer (PBS, 0.05% Tween 20) three times. Samples were diluted in Assay Diluent (PBS, 0.5% BSA, 0.05% Tween 20, 15 ppm Proclin), added to the plate, incubated 2 h at room temperature, and the plate was washed with 100 µl washing buffer six times. Biotinylated polyclonal goat anti-human MSP (R&D Systems) was diluted in Assay Diluent to 100 ng/ml, added to plate, incubated 1 h at room temperature, and plate was washed with 100 µl washing buffer six times. Amdex streptavidin-HRP (GE Healthcare) was diluted in Assay Diluent to 50 ng/ml, added to the plate, incubated 30 min at room temperature, and the plate was washed with 100 µl washing buffer six times. Binding was read by incubation with TMB/H_2_O_2_ substrate (KPL, Inc) for 15 min, addition of 1 M H_3_PO_4_, and measurement of the absorbance at 450 nm (OD_450 nm_). The MSP concentration was calculated by comparison to standard curves of MSP 689R, MSP 689C, scMSP 689R, and scMSP 689C that were titrated from 2 to 0.0082 ng/ml in 2.5-fold increments.

### Homology model of MSP β bound to RON Sema/PSI

A homology model of MSP β bound to human RON Sema/PSI was made using the coordinates from the protein data bank for MSP β (2ASU) [43], for RON Sema/PSI (4FWW) [44], and the Met Sema/PSI in complex with HGF β (1SHY) [45]. RON and MSP β were globally aligned to the Met/HGF β complex using PyMOL [46], which was used to show all structures. MSP β residue 689 was mutated to a cysteine using PyMOL.

### Anoikis assay

Assay was performed using the CytoSelect™ 96-Well Anoikis Assay (Cell Biolabs, Inc) according to the manufacturer's protocol. Cells were seeded in medium alone, medium containing 100 nM MSP 689R, or containing 100 nM MSP 689C. At 24 h, viability was assayed using the 3-(4,5-dimethylthiazol-2-yl)-2,5-diphenyltetrazolium bromide (MTT) assay and measuring the absorbance at 570 nm (OD_570 nm_).

### Statistical analysis

All data are presented as mean +/− standard deviation (SD). Statistical analysis was performed using Prism 6 (Graphpad Software). Significance was evaluated using a one-way ANOVA test followed by Tukey's multiple comparison correction. Probability values of less than 0.05 were considered statistically significant.

## Results

### RON is primarily expressed by epithelial cells in human intestine

The MSP 689C polymorphism has been hypothesized to increase IBD risk by interfering with RON-mediated inhibition of macrophage activity [7], [33], [34]. However, while RON expression and inhibitory activity on macrophages has been clearly demonstrated [24]–[28], these reports have primarily relied on studies using murine peritoneal macrophage populations that may have limited relevance to IBD. Thus, in order to identify cell types likely to be affected by alterations in MSP activity stemming from the 689C polymorphism, we sought to characterize RON expression in a variety of murine and human cell types.

**Figure 1:**
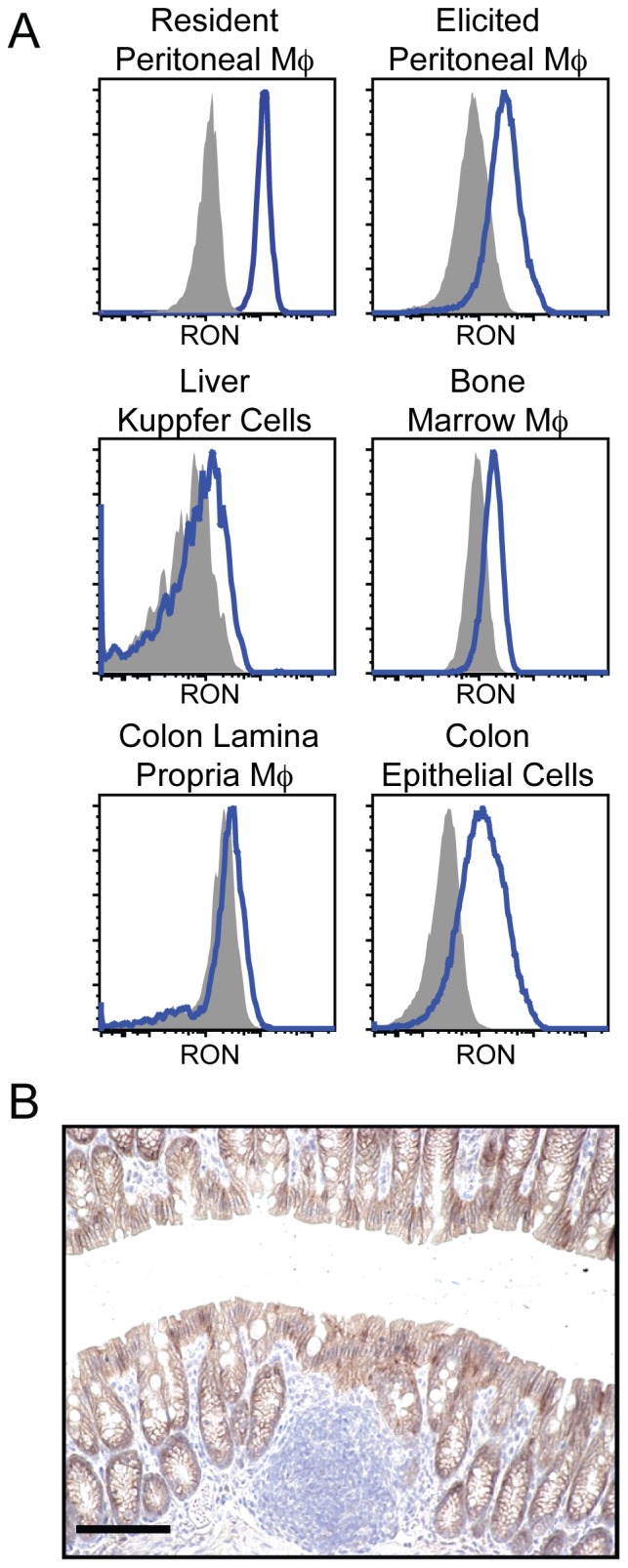
RON is expressed by myeloid and epithelial cell populations in mice. (A) Single cell suspensions of the indicated cell populations or tissues were analyzed by flow cytometry for RON expression using a monoclonal antibody specific for murine RON (blue histogram) or an isotype control antibody (shaded histogram). Macrophage populations were gated as CD45^+^F4/80^+^CD11b^+^MHC class II^+^ for *ex vivo*-derived cells and F4/80^+^CD11b^+^ for bone marrow cultured cells. Colonic epithelial cells were gated as CD45^−^ECadherin^+^. B) Representative image from IHC analysis of RON expression (brown) on tissue sections from normal mouse colon. Scale bars  =  100 µm. Data are representative of at least three similar experiments.

We first examined RON expression patterns in mice utilizing flow cytometry. Consistent with prior reports [16], resident and thioglycollate-elicited peritoneal macrophages expressed high levels of the RON receptor. Lower levels of RON were also found on other murine macrophage populations, including liver Kupffer cells and *in vitro* cultured bone marrow-derived macrophages (BMMs) [47]. We also examined RON expression on cells isolated from disassociated colon tissue. Here, we observed only weak staining for RON on the lamina propria macrophage population, but relatively high staining on colon epithelial cells (Figure 1A). To further define RON expression patterns in intestinal tissue, we performed immunohistochemistry (IHC) staining on tissue sections from murine colon. Here, we observed robust RON staining on the intestinal epithelium, but minimal staining in the lamina propria (Figure 1B).

**Figure 2:**
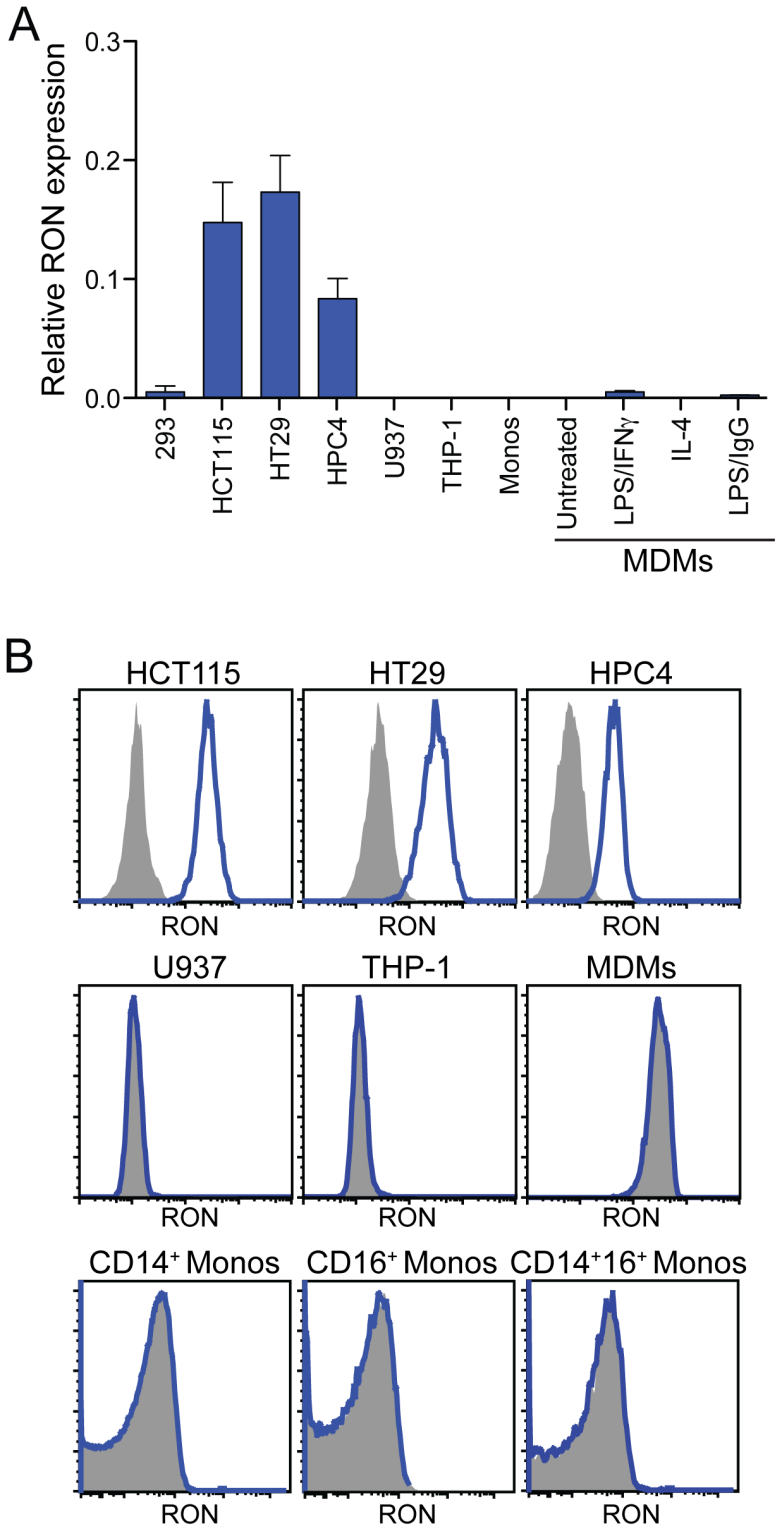
RON is preferentially expressed by human epithelial cells. (A) Quantitative RT-PCR survey of RON expression in human cell lines and primary cells. Columns 1: HEK293 control, columns 2–4: epithelial cell lines, columns 5–6: monocytic cell lines, column 7: human CD14^+^ monocytes (monos), columns 8–11: monocyte-derived macrophages (MDMs) left untreated or treated with the indicated stimuli. Data in columns 1-7 are the mean +/− SD of three independent samples and columns 8–11 are the mean of two donors. (B) Analysis of single-cell suspensions for RON expression by flow cytometry. Cells were stained with a monoclonal antibody specific for human RON (blue histogram) or an isotype control antibody (shaded histogram). MDMs were gated as CD14^+^CD33^+^.

Having recapitulated published findings on RON expression in mice, we next examined the expression of RON across different human cell types and tissues under both steady-state and inflammatory conditions. We observed substantially higher levels of RON transcripts in multiple human epithelial cell lines, compared to human myeloid cell populations that included primary CD14^+^ monocytes, monocyte-derived macrophages (MDMs), and monocytic cell lines. Treatment of MDM cultures with various stimuli, such as interferon-γ, LPS, or IL-4 failed to induce substantial upregulation of RON (Figure 2A). These data are consistent with flow cytometric analysis demonstrating RON protein expression on the surface of epithelial cells but not myeloid populations (Figure 2B) and with previously published findings demonstrating that RON can be detected on a variety of human epithelial cell lines but not peripheral blood monocytes [48].

**Figure 3:**
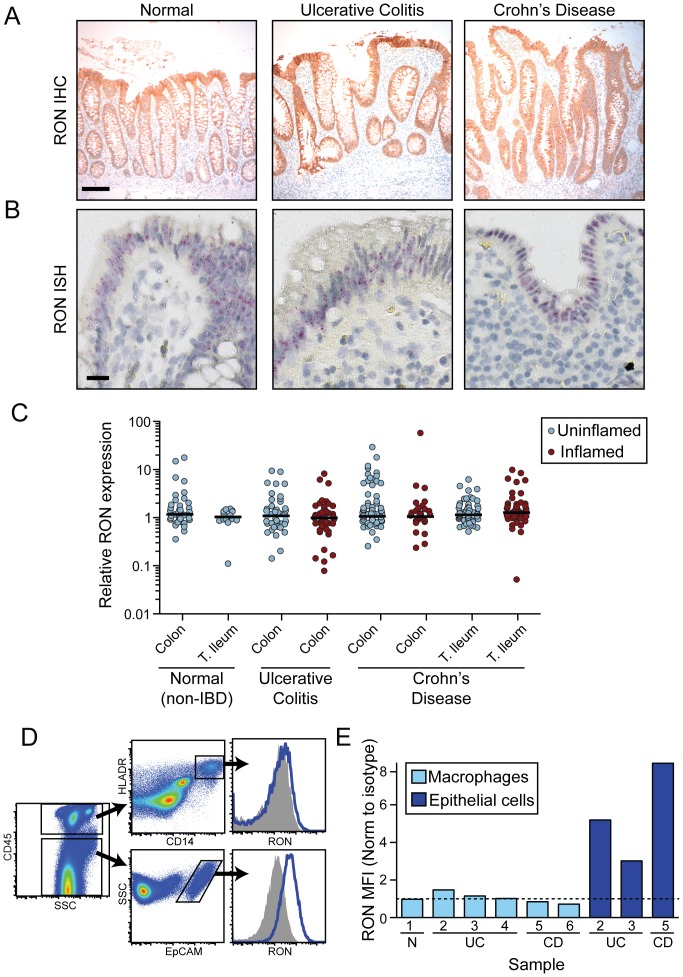
RON is preferentially expressed by epithelial cells in human tissues under steady-state and disease conditions. (A) Representative images from IHC analysis of RON protein expression (brown) on tissue sections from normal, UC, and CD colons. Scale bar  =  100 µm. (B) Representative images from ISH analysis of RON transcript expression (red) on tissue sections from normal, UC, and CD colons. Scale bar  =  20 µm. (C) Quantitative RT-PCR analysis of RON expression in human intestinal biopsies samples taken from normal individuals and uninflamed and inflamed regions of UC and CD patients. (D) Analysis of RON expression in single cell suspensions of resected intestinal tissues from an ulcerative colitis patient by flow cytometry using a monoclonal antibody specific for human RON (blue histogram) or an isotype control antibody (shaded histogram). Macrophages were gated as CD45^+^CD14^+^HLADR^+^ and epithelial cells were gated as CD45^−^EpCAM^+^ cells. (E) Quantification of RON expression by flow cytometry on macrophages and epithelial cells from resected intestinal tissue of multiple donors (macrophages: 1 non-IBD (N), 3 UC, 2 CD; epithelial cells: 2 UC, 1 CD). Patient numbers are indicated (Table S1). Data represents the mean fluorescence intensity (MFI) ratio of RON staining to isotype control staining. Dashed line indicates a ratio of 1∶1, *i.e.* lack of RON expression. N, non-IBD.

While the above data suggest that RON is not expressed under normal conditions in peripheral human monocyte/macrophage populations, these expression patterns could differ in the intestine and in the setting of IBD. IHC analysis of RON expression in normal (n = 8), UC (n = 11), and CD (n = 9) tissues demonstrated predominant localization to epithelial cells (Figure 3A and Table S1). Consistent with these data, in situ hybridization (ISH) analysis of RON transcript localization in the same panel of tissues revealed robust RON expression in the epithelium and undetectable expression in the lamina propria (Figures 3B and S1). Notably, RON expression did not substantially vary between normal and disease tissue, either by IHC, ISH, or by quantitative analysis of RON transcripts in intestinal biopsies obtained from normal, UC, and CD patients (Figures 3A, B, and C). These later data are consistent with RON being expressed by epithelial cells but not a recruited population of inflammatory cells associated with inflamed biopsy samples. To further define RON expression patterns in IBD tissue we examined single cell suspensions of human resected intestinal samples obtained from control and IBD patients by flow cytometry, first ensuring that the tissue processing and enzymatic digestion procedures did not affect RON staining (Figure S2). While only a limited number of intestinal resection samples were available for flow cytometric analysis, these data are consistent with our earlier observations, showing robust RON expression on intestinal epithelial cells and minimal expression on lamina propria-derived macrophage populations (Figures 3D and E). These findings indicate that in mice and humans, RON is highly expressed by epithelial, but not myeloid, cell populations in the intestine and suggest that the MSP risk allele may confer increased susceptibility to IBD through effects on the epithelium.

### MSP 689R and 689C variants bind RON with similar affinities

**Figure 4:**
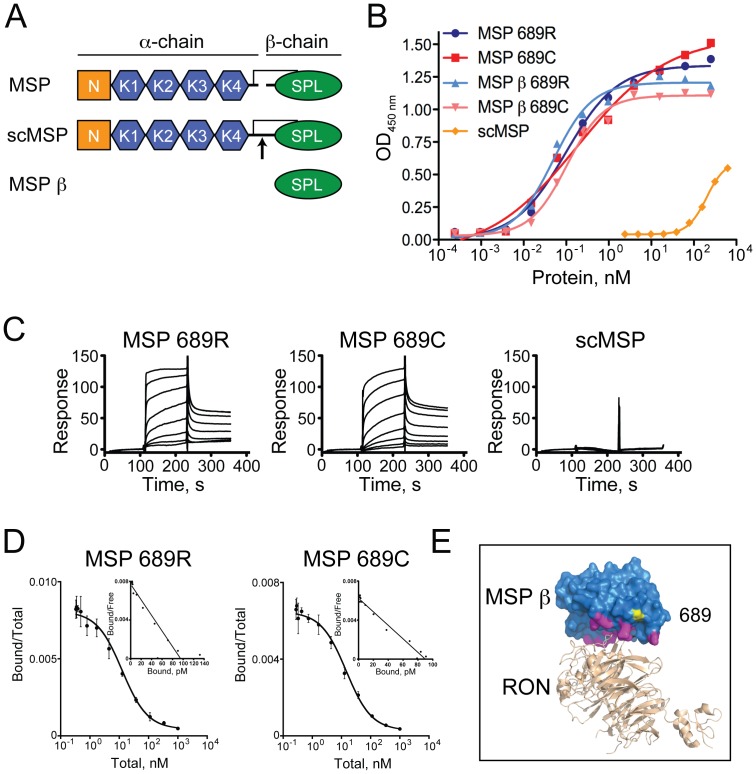
The 689C polymorphism in MSP does not affect binding to RON. (A) Overview of recombinant human MSP proteins used in this study. MSP α- and β-chains, PAN domain (N), kringle domains (K), serine protease-like domain (SPL) are indicated. scMSP is inactive due to the R483E mutation at the proteolytic cleavage site (arrow). 689R and 689C versions of all MSP proteins were generated. (B) Cell-free binding assay consisting of plate bound RON-Fc and soluble MSP. Means of three replicates per group are shown. Lines represent dose-response curves fit to a 4 parameter equation, which yielded EC_50_ values of 0.1, 0.2, 0.05 and 0.1 nM for MSP 689R, 689C, MSP β 689R and MSP β 689C, respectively. (C) SPR analysis of MSP proteins binding to immobilized RON Sema/PSI, showing relative response in response units. Data are representative of three independent experiments. (D) Radioligand binding assay of MSP binding to RON. Competition binding to 3T3-hRON cells (large graphs) used to generate affinities and Scatchard plots (inset graphs) are shown. Mean +/− SD of three independent experiments is shown. (E) Homology model of the structure of RON Sema/PSI (PDB code 4FWW) in beige bound to MSP β (PDB code 2ASU) in blue. RON and MSP β were globally aligned to the Met/HGF β complex (PDB code 1SHY). Predicted locations of the MSP β contact residues within 4 Å of RON are highlighted in magenta. MSP β residue arginine 689 was mutated to a cysteine and is shown in yellow.

Having determined that epithelial cells are the likely targets of MSP-RON activity in the intestine, we next examined the consequences of the 689C polymorphism for RON activation. Several forms of recombinant MSP were expressed and purified from mammalian cells (Figure 4A), including full-length versions of the 689R and 689C MSP variants that, consistent with previously published studies, required a cysteine to alanine substitution at amino acid 672 (C672A) in order to obtain properly folded protein [34], [49]. Constitutively inactive, single-chain MSP (scMSP) proteins bearing an arginine to glutamic acid mutation at amino acid 483 (R483E) that prevents proteolytic cleavage to the active two-chain form, were also expressed and purified as controls. Finally, recombinant 689R and 689C versions of the MSP β-chain (MSP β), the domain responsible for high affinity interactions with RON [50], [51], were generated. Importantly, MSP β proteins were successfully expressed and purified without the mutation at position 672 and thus represent the wild-type sequence of this domain.

In cell-free assays of MSP binding to immobilized RON, 689R and 689C protein variants showed overlapping dose-response curves. As expected, the scMSP mutant showed minimal binding to RON-coated plates (Figure 4B). To more precisely quantify MSP interactions with RON, surface plasmon resonance (SPR) was used to determine the affinity of soluble MSP 689R and 689C to immobilized RON. Again, no significant differences in RON binding kinetics were observed between the 689R and 689C versions of either full-length MSP or MSP β and no binding was observed for scMSP, using either direct or indirect RON immobilization (Figure 4C and Table 1). Similar results were obtained using biolayer interferometry (BLI), a related technique used for real-time quantification of molecular interactions (Table 1). Notably, we observed no effect of mutating position 672 in MSP β on the ability of either the 689R or 689C variants to bind to RON (Table 1), suggesting that full-length MSP binding to RON is not influenced by the C672A mutation necessary for its expression.

**Table 1:** Binding kinetics and affinities of MSP 689R and 689C proteins to RON.

Protein	k_on_ (M^−1^s^−1^)×10^−5^	k_off_ (s^−1^)×10^2^	K_D_ (nM)^a^	K_D_ (nM)a,e,f
	b5.1	b0.33	b6.4±0.1	e12.6±1.7
MSP 689R	c2.1	c0.15	c7.1±1.1	f23.0±2.0
	d2.5	d0.25	d9.9±0.2	
	b0.17	b0.02	b9.6±0.6	e13.0±2.8
MSP 689C	c1.2	c0.12	c9.6±0.5	f25.7±6.0
	d4.3	d0.42	d9.4±0.7	
	b18.1	b1.75	b9.6±0.8	
MSP β 689R	c27.1	c1.84	c6.6±0.1	
	d4.7	d0.35	d7.5±0.2	
	b44.2	b4.19	b9.0±1.1	
MSP β 689C	c14.6	c1.34	c9.1±0.2	
	d10.5	d0.93	d9.1±1.7	
MSP β 672C 689R	b8.6	b0.66	b6.3±0.8	
MSP β 672C 689C	b7.7	b0.75	b9.7±0.7	

^a^Data are the average ± standard deviations of three or more independent determinations in general.

^b^SPR capturing RON-Fc at 25°C,

^c^SPR using immobilized RON Sema/PSI at 25°C,

^d^BLI using RON-Fc at 30°C,

^e^Radioligand binding to 3T3-hRON cells for 2 h at room temperature,

^f^Radioligand binding to 3T3-hRON cells for 4 h on ice.

To confirm the above results, we also performed a radioligand binding assay using labeled MSP 689R and 689C proteins and a fibroblast cell line engineered to express human RON (3T3-hRON). In agreement with the cell-free assays, competition binding and Scatchard analysis revealed no significant difference in the RON binding affinities between the MSP variants (Figure 4D and Table 1). Taken together, these data indicate that the 689C polymorphism does not affect the binding between MSP and RON. This conclusion is in accord with a homology model of RON bound to MSP β showing that residue 689 resides outside of the putative MSP-RON interface (Figures 4E and S3).

### pro-MSP 689R and 689C variants undergo similar proteolytic activation

**Figure 5:**
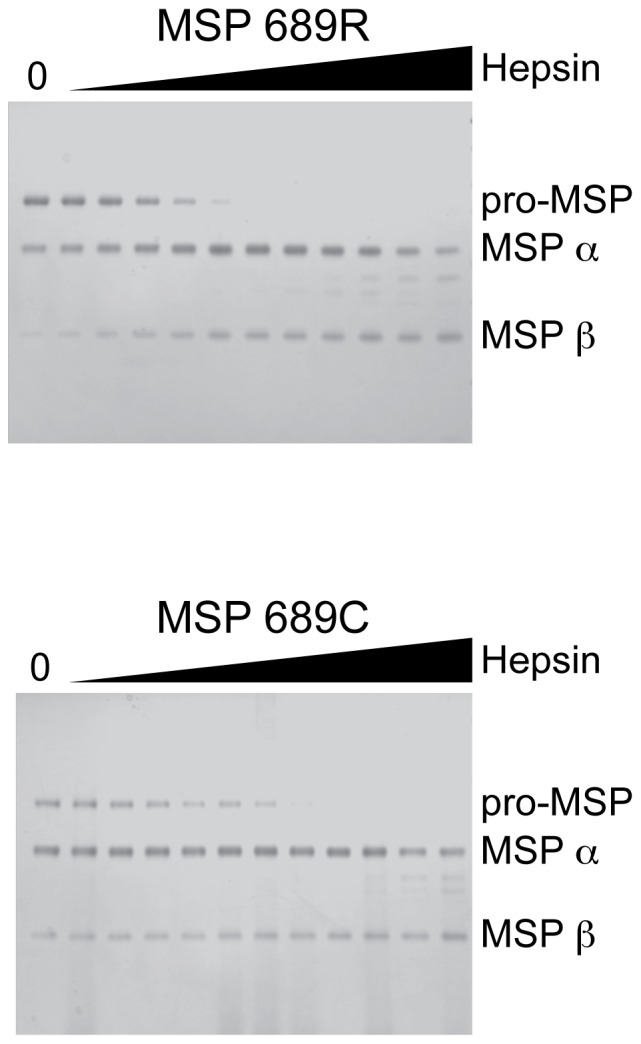
The 689C polymorphism does not affect proteolytic activation of MSP by hepsin. Purified MSP 689R (upper panel) and MSP 689C (lower panel) consisting of both pro-MSP and MSP were mixed with 100 nM to 97 pM hepsin in a 2-fold dilution series and analyzed by gel electrophoresis under reducing conditions. Relative mobilities corresponding to pro-MSP, MSP α, and MSP β are indicated. Data are representative of three independent experiments.

As proteolytic activation of MSP represents a critical regulatory point controlling RON activity *in vivo*, we examined whether this process differs between MSP 689R and 689C. Recombinant MSP exists in both inactive pro-MSP (uncleaved) and active MSP (cleaved) forms following purification from transfected cell supernatants, presumably due to endogenous protease activity present during expression. We assessed the ability of hepsin, a protease that cleaves and activates pro-MSP [15], to cleave the 689R or 689C single-chain proteins to completion. No obvious differences were observed in the concentrations of hepsin required to completely cleave these variants over a 1-hour period (Figure 5). These data suggest that the 689C polymorphism does not alter RON signaling through differential effects on proteolytic activation of pro-MSP.

### MSP 689R and 689C variants induce similar RON signaling

**Figure 6:**
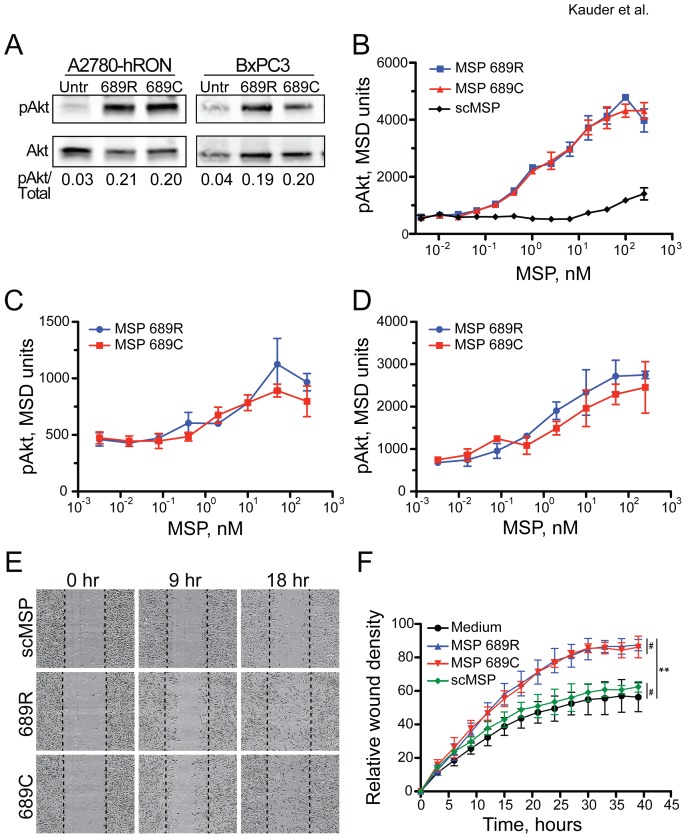
The 689C polymorphism does not affect MSP signaling through RON. (A) Western blot analysis of total Akt and pAkt in A2780-hRON and BxPC3 cells treated as indicated. Blot was performed in triplicate and mean of the pAkt/total ratios is shown. (B) Quantitation of pAkt by MSD analysis in 3T3-hRON cells treated with scMSP or MSP variants. Mean +/− SD of triplicate wells is shown. Data are representative of three independent experiments. (C) Quantification of pAkt by MSD analysis in HCT15 cells treated with MSP variants. Mean +/− SD of triplicate wells is shown. Data are representative of two independent experiments. (D) Quantitation of pAkt by MSD analysis in human primary colon cells treated with MSP variants. Mean +/− SD of triplicate wells is shown. Data are representative of two independent experiments. (E) Images from scratch wound assay of 3T3-hRON cells treated with scMSP or MSP variants. Images are from the same cell culture at the indicated times after scratch wounding. Dashed lines represent position of initial scratch. (F) Quantitation of scratch wound assay from 3T3-hRON cells treated with medium-alone, scMSP, or MSP variants. Mean +/− SD of three treatments is shown. Data are representative of three independent experiments. ^#^not significant, **p≤0.0003 for comparisons between medium and MSP variants.

While our studies revealed no difference in proteolytic activation or RON binding between the MSP variants, the 689C polymorphism could still affect MSP-dependent signal transduction through the RON receptor. To address this point, down-stream biological effects of MSP variants on several RON-expressing cell lines were determined. *In vitro* stimulation of A2780-hRON cells [38] and BxPC3 cells, a pancreatic epithelial cell line that expresses endogenous RON, with either full-length activated MSP 689R or 689C protein induced similar levels of phosphorylated Akt (pAkt) by western blot analysis (Figure 6A). Furthermore, quantitative Meso Scale Discovery (MSD) analysis of pAkt induction in 3T3-hRON cells failed to reveal any differences in the activities of MSP 689R and 689C across a wide range of doses (Figure 6B). Consistent with its lack of RON binding, scMSP did not induce a robust pAkt signal. To extend these findings to a more physiologically relevant cell type, we also performed MSD analysis of pAkt induction in two cell types that express endogenous RON, the HCT15 intestinal epithelial cell line and HPC5 human primary colon cells. Treatment of these cells with varying doses of MSP 689R and 689C revealed that the MSP dose-dependent increase in pAkt levels was not affected by the 689C polymorphism (Figure 6C and D). Given the well-validated role of the PI3-Kinase/Akt pathway in promoting cell survival, we also examined the ability of MSP 689R and 689C to protect HCT15 epithelial cells from anoikis, a form of apoptosis induced by lack of adhesion to an extracellular matrix substrate. Both variants of MSP were able to induce a small but significant increase in cell viability following plating under non-adherent conditions (Figure S4).

The above findings are consistent with numerous reports implicating RON signaling in the wound repair process through induction of epithelial cell proliferation, survival, and migration [9], [10], [14], [19]–[22]. To determine whether the 689C polymorphism affects these down-stream cellular responses to MSP, we utilized an *in vitro* cell monolayer scratch-wounding model and evaluated wound closure in the presence or absence of MSP variants. Scratch wounds were made in confluent monolayers of 3T3-hRON cells and cultures were subsequently treated with MSP 689R, MSP 689C, medium alone, or inactive scMSP. Treatment with MSP 689R and 689C resulted in similar recovery from wounding that was greater than the untreated and scMSP-treated cultures (Figures 6E and F). These MSP-induced responses were dependent on RON expression, as no effects of treatment were observed in RON-negative 3T3 cells (Figure S5). Taken together, these data indicate that the 689C polymorphism does not alter the ability of MSP to bind to, signal through, or elicit functional responses from the RON receptor. In addition, they confirm the ability of MSP-dependent RON signaling to drive cellular responses that are relevant for wound repair.

### Carriers of the MSP 689C polymorphism have reduced quantities of serum MSP

**Figure 7:**
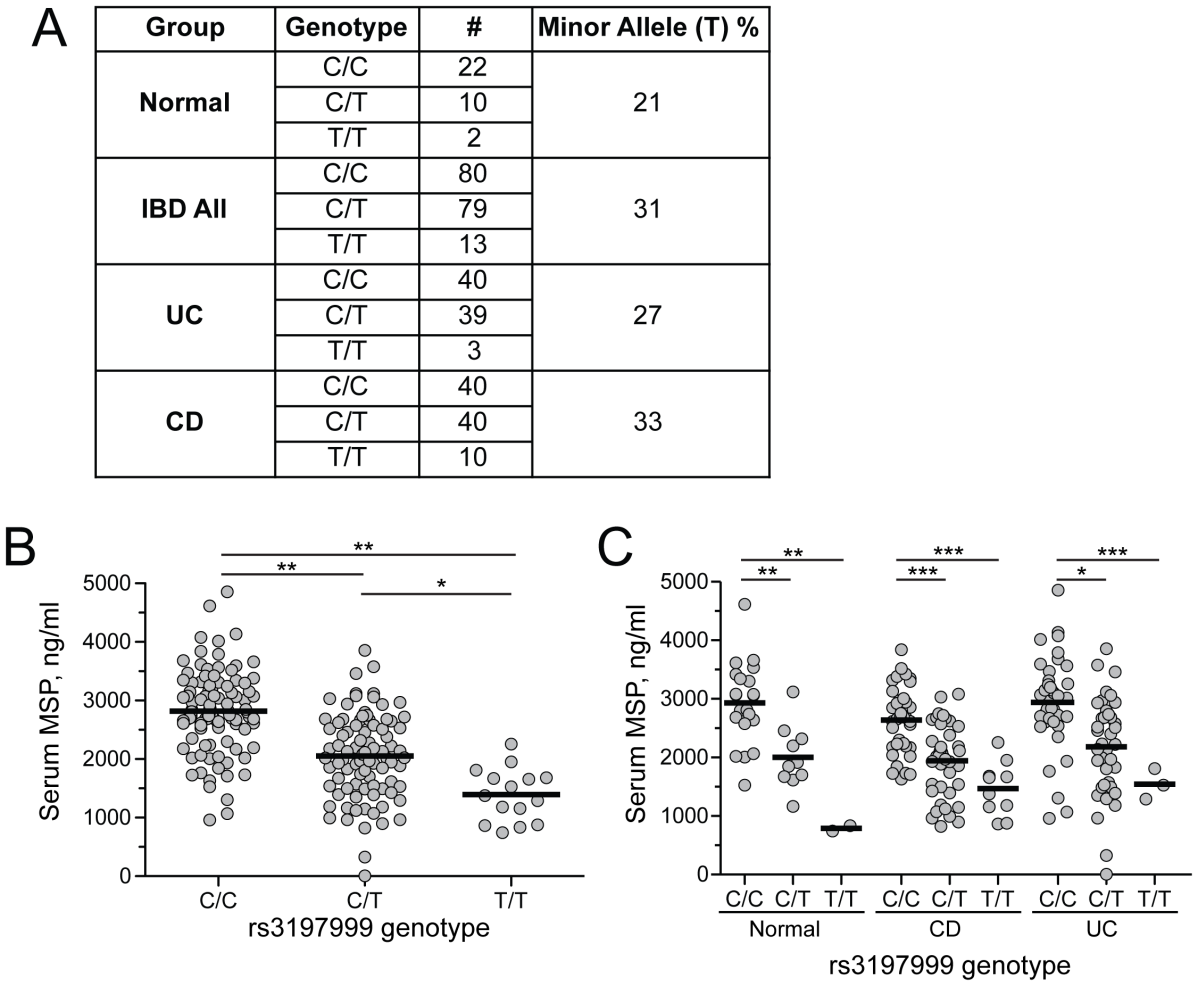
Carriers of the 689C polymorphism have lower quantities of serum MSP. (A) Analysis of rs3197999 alleles in DNA samples from normal, UC, and CD patients. The C allele encodes MSP 689R and T allele encodes MSP 689C. Allele frequency and number are indicated for each group. (B) MSP concentrations in matched serum samples of EMBARK subjects determined by ELISA, with results grouped by rs3197999 genotype. *p<0.005, **p<0.0005. (C) Serum MSP concentrations grouped by genotype and disease status. *p<0.05, **p<0.005, ***p<0.0005.

MSP is predominately expressed by the liver, where it is secreted into the serum and circulates at relatively high concentrations [52]. Given our inability to identify differences in the function of MSP variants for processes proximal to RON binding and activation, we next looked further upstream, hypothesizing that the 689C polymorphism may affect quantities of circulating MSP. Matched human serum and DNA samples from 204 donors were analyzed for both the rs3197999 genotype and MSP serum concentrations. Consistent with previous genetic studies [2]–[7], the minor allele of MSP was enriched in UC and CD patients compared to individuals without disease (Figure 7A). However, given the modest contribution of this allele to overall IBD risk and the relatively small size of our study group compared to GWAS cohorts, these results failed to meet statistical significance.

To establish the relationship between rs3197999 genotype and serum MSP concentrations, we developed an ELISA assay to measure MSP in human serum. Importantly, this assay had equal sensitivity for detecting MSP 689R and MSP 689C proteins and was unaffected by the activation state of the protein (Figure S6). Analysis of serum MSP concentrations in the rs3197999 genotyped cohort revealed that heterozygous (C/T) carriers of the MSP 689C polymorphism had 27 percent and homozygous (T/T) carriers had 50 percent lower quantities of MSP compared to individuals carrying two copies of MSP 689R variant (Figure 7B). Importantly, these decreased concentrations of serum MSP were not a secondary effect of IBD, as normal individuals and patients diagnosed with CD or UC showed similar genotype-dependent reductions in MSP concentrations (Figure 7C). Taken together, these data indicate that the 689C polymorphism is associated with decreased levels of circulating MSP and suggest that the resulting reduction in RON pathway activity may impact the efficiency of wound repair in intestinal epithelial cells.

## Discussion

The identification of a non-synonymous coding variant in MSP that can confer increased risk of developing IBD has suggested a role for RON in intestinal homeostasis. However, as with many disease-associated risk alleles, an incomplete understanding of the functional consequences of this polymorphism has limited our ability to decipher the contributions of the MSP-RON pathway to the pathogenesis of IBD. Here we find that, contrary to previous reports, the 689C polymorphism does not affect MSP binding to RON or subsequent down-stream signaling. Instead, this variant is associated with decreased quantities of circulating MSP in the serum of both normal individuals and IBD patients, suggesting that reduced MSP-RON pathway activity due to limited ligand availability may underlie the genetic association with IBD susceptibility.

Previous studies have demonstrated that the RON receptor plays an important role in regulating the production of pro-inflammatory mediators from murine macrophages [24]–[28], leading to speculation that the increased genetic risk for IBD associated with the 689C polymorphism may stem from dysregulated macrophage function [7], [33]–[35]. We confirmed that RON is highly expressed by some murine macrophage populations, although levels of expression were dependent on the tissue of origin, with intestinal macrophages expressing minimal receptor compared to peritoneal macrophages or intestinal epithelial cells. Consistent with these expression patterns in mice, we found that human intestinal macrophage populations do not express substantial levels of RON receptor. Rather, consistent with previously published observations [17], RON was found to be constitutively and highly expressed by the intestinal epithelium in humans. Importantly, this expression pattern did not appreciably change under conditions of inflammation due to IBD. While our data point to the epithelial compartment as being the primary target of MSP activity and RON signaling in the intestine, they do not exclude the possibility that RON is expressed in human macrophage populations that reside in other tissues, such as the lung and peritoneum [53].

In order to maintain the integrity of the intestinal epithelial barrier, multiple pathways have evolved to promote restitution of the epithelium following injury. Indeed, polymorphisms in genes likely involved in maintaining epithelial barrier integrity, including CDH1, GNA12, PTGER4, NKX2-3, and STAT-3, confer increased risk for IBD [33]. Our study places the MSP 689C polymorphism within this same group of epithelial biology-related risk alleles and suggests that diminished function in the MSP-RON pathway, due to decreased ligand availability, leads to an impaired ability to repair or maintain an intact epithelial barrier. Notably, our findings have implications for diseases other than IBD. The 689C polymorphism in MSP also confers increased risk for primary sclerosing cholangitis [54], [55]. RON is highly expressed by multiple epithelial compartments, thus raising the possibility that defects in the MSP-RON pathway may impact cholangiocyte homeostasis and the ability of the biliary epithelial barrier to protect the liver parenchyma from toxic products within bile.

Our findings demonstrating that the 689C polymorphism does not affect the kinetics of MSP binding to the RON receptor differ from those suggesting that the 689C polymorphism decreases the affinity of MSP for RON [34]. The reason for this discrepancy is not known, but could relate to differences in the MSP protein used. While we evaluated the effects of the 689C polymorphism on binding of full length MSP to RON, the previous study utilized a truncated version of MSP consisting of MSP β and the C-terminal 19 residues of MSP α. This non-natural protein construct may have altered RON binding properties compared to full-length MSP and, as it lacked the complete MSP α-chain, could not be used to examine RON signaling or cellular activation. Another more recent publication has reported that the 689C polymorphism actually enhances the ability of MSP to induce chemotaxis and proliferation of the human monocytic cell line, THP-1, which was found to express low levels of RON protein by western blot [35]. We could not confirm expression of RON on the surface of THP1 cells by FACS analysis. In addition, this study relied on supernatants from cells transfected with 689R and 689C rather than purified MSP proteins, raising the possibility that factors present within the transfected cell culture medium could have influenced THP1 responses, independent of MSP.

The mechanism by which the 689C polymorphism decreases MSP quantities in the serum remains to be fully elucidated. Previous studies have suggested that hepatic catabolism is a primary clearance mechanism for circulating pro-MSP [56], thus raising the possibility that the 689C variant is differentially absorbed or processed by the liver. However, we cannot rule out effects of the rs3197999 SNP on rates of transcription from the endogenous MSP locus or subsequent RNA processing. Alternatively, the rs3197999 variant may be in linkage disequilibrium with additional genetic variants, such as promoter polymorphisms, that are directly responsible for decreasing production of MSP and subsequent secretion into circulation.

The finding that the MSP 689C polymorphism confers increased genetic risk for IBD, combined with our data identifying the functional consequences of this polymorphism and the likely cellular compartment impacted, suggest that the MSP-RON pathway is important for maintaining epithelial barrier integrity. This conclusion raises the intriguing possibility that therapeutic strategies designed to stimulate RON activity could promote wound repair in the context of IBD.

## Supporting Information

Figure S1 **Specificity of ISH probe used to detect RON in human tissues.** Representative images from serial sections of colon tissue from an ulcerative colitis patient hybridized with a probe specific to *Bacillus subtilis* dihydropicolinate reductase (dapB) as a negative control (left) or a probe specific for RON (right).
(TIF)

Figure S2 **Detection of human RON by flow cytometry is not affected by enzymatic digestion protocols used to generate single cell suspensions from intestinal resections.** Analysis of RON expression in human primary colon cells by flow cytometry. Cells were either untreated (left panel) or treated with digestion conditions identical to those used to generate single cell suspensions of resected human intestine. Cells were stained with a monoclonal antibody specific for human RON (blue histogram) or an isotype control antibody (shaded histogram).
(TIF)

Figure S3 **Homology model of the structure of MSP β bound to RON Sema/PSI based on the crystal structure of HGF β bound to Met Sema/PSI.** Homology model of the structure of RON Sema/PSI (PDB code 4FWW) in beige bound to MSP β (PDB code 2ASU) in blue. RON Sema/PSI and MSP β were globally aligned to the complex (PDB code 1SHY) of Met Sema/PSI (pale green) with HGF β (pink). The sulfur atom of MSP β 689C is shown as a yellow sphere. Residues E221 of Met and A223 of RON sit on top of the pseudo S1 pocket of HGF β and MSP β, respectively and are shown as sticks.
(TIF)

Figure S4 **Both MSP 689C and 689R can protect the anchorage-dependent epithelial cell line HCT15 from anoikis.** MTT assay for cell viability after overnight culture on anchorage-resistant plates in the presence of medium (black bar), MSP 689R (blue bar), or MSP 689C (red bar). Mean of three treatments is shown and error bars indicate standard deviation. Data are from one experiment. ^#^not significant, *p<0.05.
(TIF)

Figure S5 **MSP variants do not induce scratch wound closure in parental 3T3 cells that do not express RON.** Quantitation of scratch wound assay from parental 3T3 cells treated with scMSP or MSP variants. Mean of three treatments is shown and error bars signify standard deviation. Data are representative of three independent experiments.
(TIF)

Figure S6 **ELISA assay for determining the concentrations of human MSP in serum.** MSP ELISA assay on recombinant human MSP variants and scMSP that were first titrated to indicated concentrations. Error bars represent standard deviation.
(TIF)

Table S1 **Human intestinal tissue used for analysis of RON expression.**
(PDF)
