# Supplementary figures and images for: Functional Consequences of the Macrophage Stimulating Protein 689C Inflammatory Bowel Disease Risk Allele
Source: PLoS One. 2013 Dec 23;8(12):e83958. doi: 10.1371/journal.pone.0083958 (PMC3884107; doi:10.1371/journal.pone.0083958)

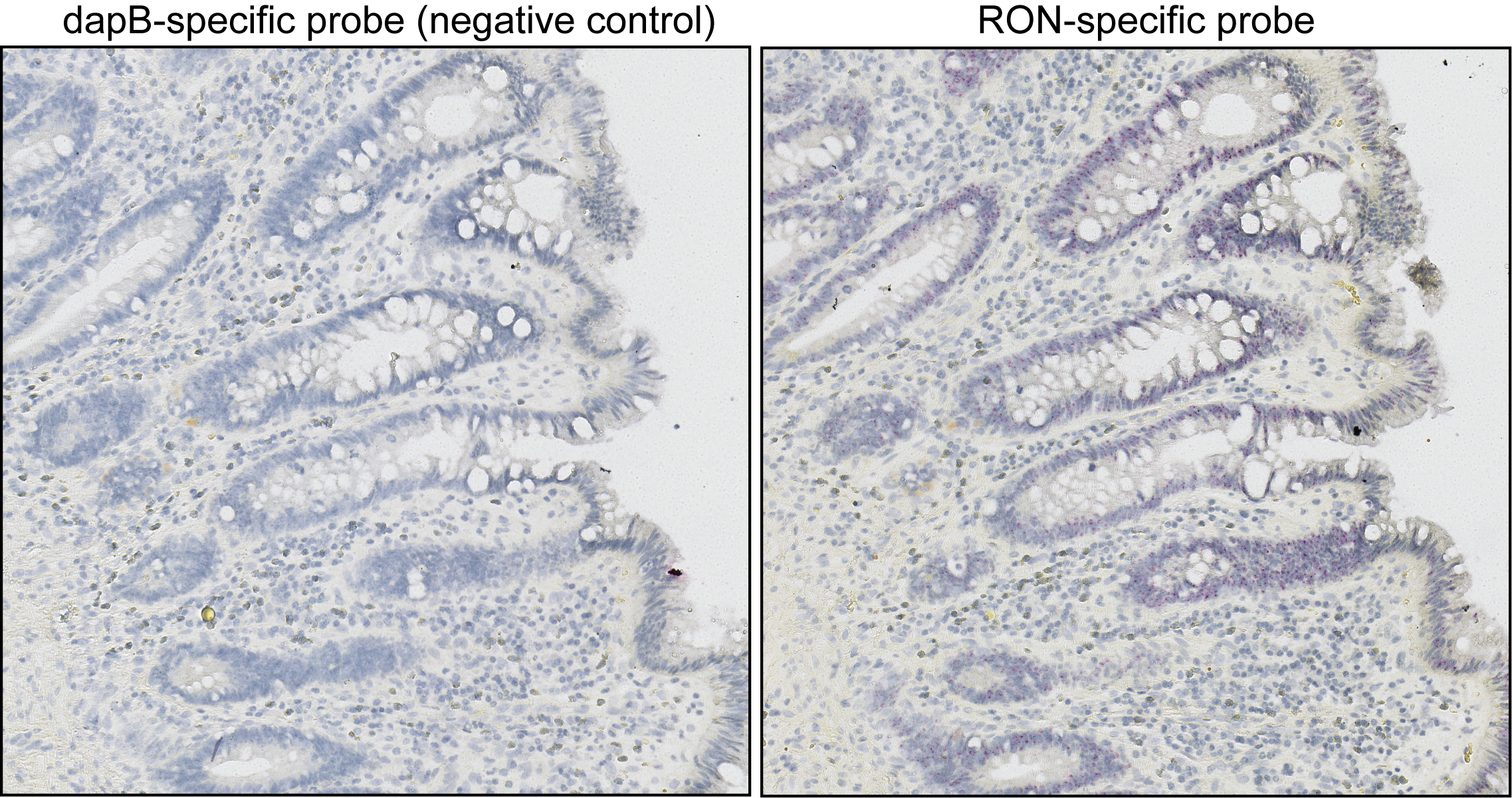

Supplement: Figure S1 — Specificity of ISH probe used to detect RON in human tissues. Representative images from serial sections of colon tissue from an ulcerative colitis patient hybridized with a probe specific to Bacillus subtilis dihydropicolinate reductase (dapB) as a negative control (left) or a probe specific for RON (right). (TIF) [file pone.0083958.s001.tif]

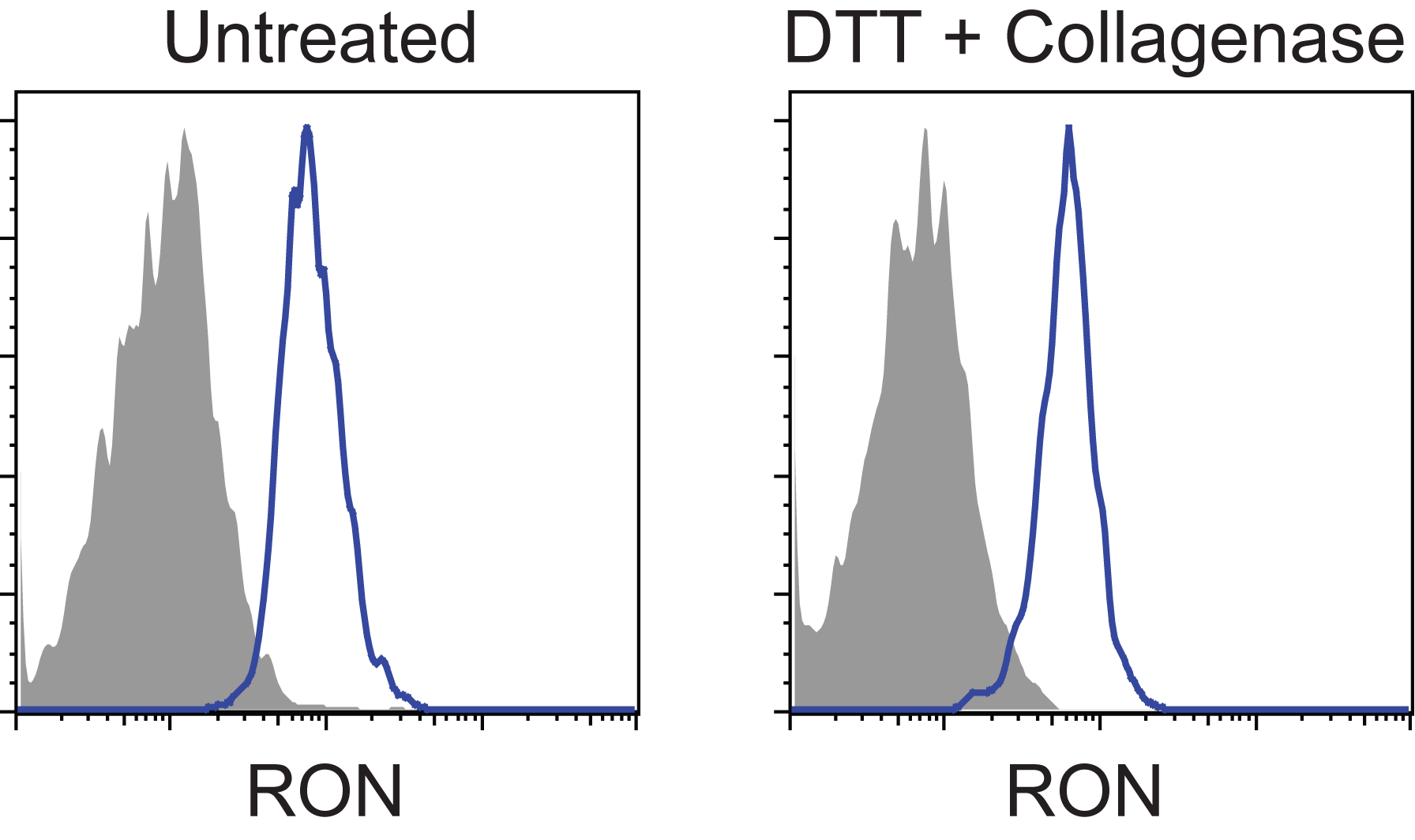

Supplement: Figure S2 — Detection of human RON by flow cytometry is not affected by enzymatic digestion protocols used to generate single cell suspensions from intestinal resections. Analysis of RON expression in human primary colon cells by flow cytometry. Cells were either untreated (left panel) or treated with digestion conditions identical to those used to generate single cell suspensions of resected human intestine. Cells were stained with a monoclonal antibody specific for human RON (blue histogram) or an isotype control antibody (shaded histogram). (TIF) [file pone.0083958.s002.tif]

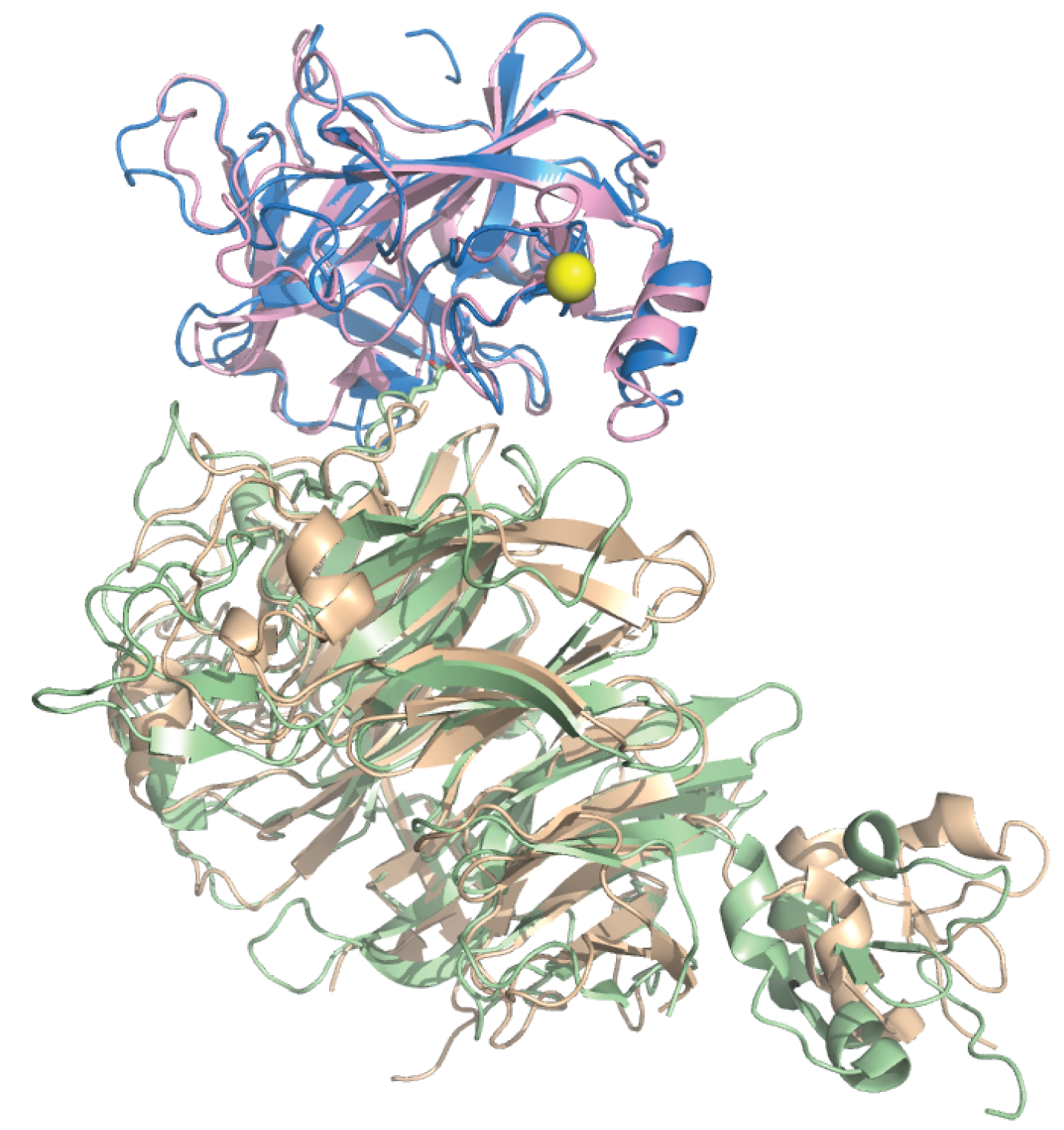

Supplement: Figure S3 — Homology model of the structure of MSP β bound to RON Sema/PSI based on the crystal structure of HGF β bound to Met Sema/PSI. Homology model of the structure of RON Sema/PSI (PDB code 4FWW) in beige bound to MSP β (PDB code 2ASU) in blue. RON Sema/PSI and MSP β were globally aligned to the complex (PDB code 1SHY) of Met Sema/PSI (pale green) with HGF β (pink). The sulfur atom of MSP β 689C is shown as a yellow sphere. Residues E221 of Met and A223 of RON sit on top of the pseudo S1 pocket of HGF β and MSP β, respectively and are shown as sticks. (TIF) [file pone.0083958.s003.tif]

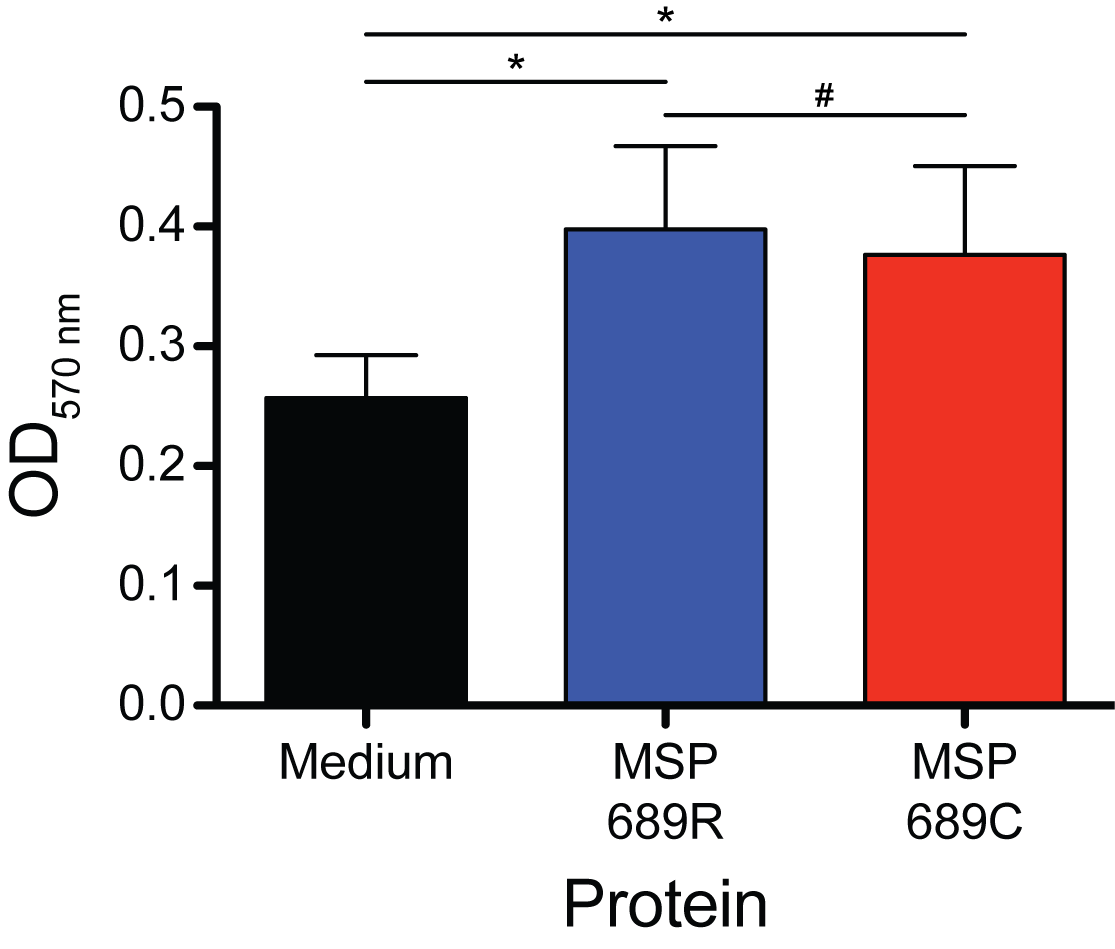

Supplement: Figure S4 — Both MSP 689C and 689R can protect the anchorage-dependent epithelial cell line HCT15 from anoikis. MTT assay for cell viability after overnight culture on anchorage-resistant plates in the presence of medium (black bar), MSP 689R (blue bar), or MSP 689C (red bar). Mean of three treatments is shown and error bars indicate standard deviation. Data are from one experiment. #not significant, *p<0.05. (TIF) [file pone.0083958.s004.tif]

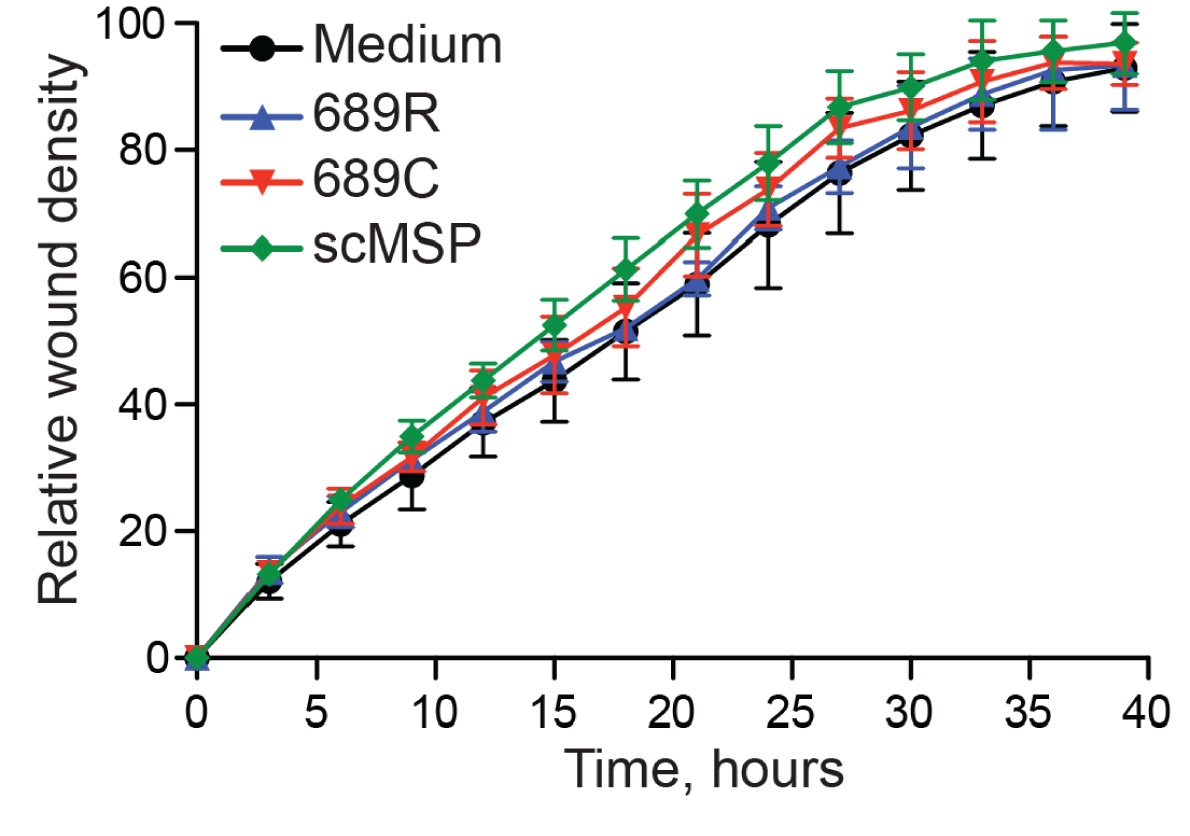

Supplement: Figure S5 — MSP variants do not induce scratch wound closure in parental 3T3 cells that do not express RON. Quantitation of scratch wound assay from parental 3T3 cells treated with scMSP or MSP variants. Mean of three treatments is shown and error bars signify standard deviation. Data are representative of three independent experiments. (TIF) [file pone.0083958.s005.tif]

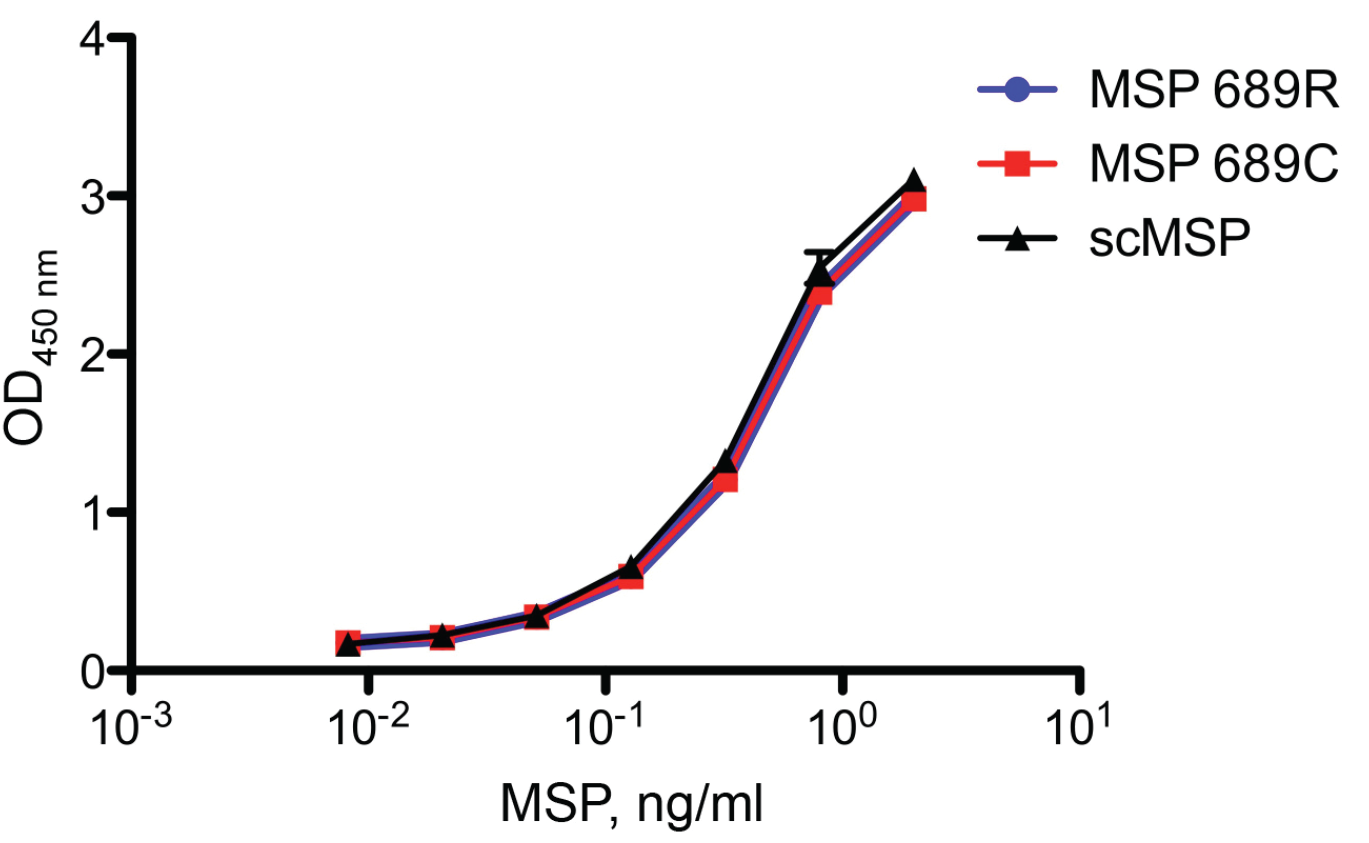

Supplement: Figure S6 — ELISA assay for determining the concentrations of human MSP in serum. MSP ELISA assay on recombinant human MSP variants and scMSP that were first titrated to indicated concentrations. Error bars represent standard deviation. (TIF) [file pone.0083958.s006.tif]
