# Supplementary material for: Functional Consequences of the Macrophage Stimulating Protein 689C Inflammatory Bowel Disease Risk Allele
Source: PLoS One. 2013 Dec 23;8(12):e83958. doi: 10.1371/journal.pone.0083958 (PMC3884107; doi:10.1371/journal.pone.0083958)
Supplement: Table S1 — Human intestinal tissue used for analysis of RON expression. (PDF) [file pone.0083958.s007.pdf]

Table S1. Human intestinal tissue used for analysis of RON expression

Patient	Cohort	Resected Tissue	Assay
1	Control (Diverticulitis)	Descending colon	Flow
2	UC	Ascending, transverse colon	Flow
3	CD	Ascending, transverse colon	IHC, ISH, Flow
4	UC	Subtotal colectomy	Flow
5	CD	Subtotal colectomy	IHC, ISH, Flow
6	CD	Terminal ileum	Flow
7	CD	Ileum, ascending, transverse, and descending colon	IHC, ISH
8	UC	Ileoanal anastomosis, laproscopic proctocolectomy	IHC, ISH
9	UC	35 cm colon to analverge	IHC, ISH
10	UC	4.0 cm of ileum, 75.0 cm of colon	IHC, ISH
11	UC	Total proctocolectomy, ileostomy	IHC, ISH
12	UC	Subtotal colectomy	IHC, ISH
13	Control (Cancer)	10cm proximal and distal to tumor	IHC, ISH
14	Control (Cancer)	Anterior section of sigmoid colon	IHC, ISH
15	Control (Cancer)	Right hemicolectomy	IHC, ISH
16	Control (Cancer)	Right hemicolectomy, terminal ileum	IHC, ISH
17	CD	Abd colectomy, end ileostomy	IHC, ISH
18	UC	Colon	IHC, ISH
19	Control (Cancer)	Right colon hemicolectomy, 5.3 cm of terminal ileum	IHC, ISH
20	UC	Total proctocolectomy, end ileostomy	IHC, ISH
21	CD	Ileum and colon	IHC, ISH
22	UC	Distal sigmoid colon	IHC, ISH
23	CD	Colon	IHC, ISH
24	CD	Colon and terminal ileum	IHC, ISH
25	UC	2.5 cm Ileum, 85 cm colon	IHC, ISH
26	UC	Colon	IHC, ISH
27	UC	Entire colon	IHC, ISH
28	Normal	na	IHC, ISH
29	Control (Diverticulitis)	na	IHC, ISH
30	CD	na	IHC, ISH
31	CD	Colon	IHC
32	Control	Colon	ISH
